# Urinary podocyte mRNAs precede microalbuminuria as a progression risk marker in human type 2 diabetic nephropathy

**DOI:** 10.1038/s41598-020-75320-1

**Published:** 2020-10-23

**Authors:** Akihiro Fukuda, Akihiro Minakawa, Masao Kikuchi, Yuji Sato, Masanao Nagatomo, Shuji Nakamura, Tetsu Mizoguchi, Naoya Fukunaga, Hirotaka Shibata, Abhijit S. Naik, Roger C. Wiggins, Shouichi Fujimoto

**Affiliations:** 1 Department of Endocrinology, Metabolism, Rheumatology and Nephrology, Faculty of Medicine, Oita University, 1-1 Idaigaoka, Hasama-machi, Yufu, Oita, 879-5593 Japan; 2 Division of Nephrology, Department of Internal Medicine, Faculty of Medicine, University of Miyazaki, Miyazaki, Japan; 3 Heiwadai Hospital, Miyazaki, Japan; 4 JCHO Nankai Medical Center, Oita, Japan; 5 Division of Nephrology, Department of Internal Medicine, University of Michigan, Ann Arbor, MI USA; 6 Department of Hemovascular Medicine and Artificial Organs, University of Miyazaki, Miyazaki, Japan

**Keywords:** Biomarkers, Endocrinology, Nephrology

## Abstract

Earlier detection of progression risk in diabetic nephropathy will allow earlier intervention to reduce progression. The hypothesis that urinary pellet podocyte mRNA is a more sensitive progression risk marker than microalbuminuria was tested. A cross sectional cohort of 165 type 2 diabetics and 41 age and sex-matched controls were enrolled. Podocyte stress (Urinary pellet podocin:nephrin mRNA ratio), podocyte detachment (Urinary pellet podocin mRNA:creatinine ratio: UPPod:CR) and a tubular marker (Urinary pellet aquaporin 2:creatinine ratio) were measured in macro-albuminuric, micro-albuminuric and norm-albuminuric groups. eGFR was reassessed after 4 years in 124 available diabetic subjects. Urinary pellet podocyte and tubular mRNA markers were increased in all diabetic groups in cross-sectional analysis. After 4 years of follow-up univariable and multivariate model analysis showed that the only urinary markers significantly related to eGFR slope were UPPod:CR (*P* < 0.01) and albuminuria (*P* < 0.01). AUC analysis using K-fold cross validation to predict eGFR loss of ≥ 3 ml/min/1.73m^2^/year showed that UPPod:CR and albuminuria each improved the AUC similarly such that combined with clinical variables they gave an AUC = 0.70. Podocyte markers and albuminuria had overlapping AUC contributions, as expected if podocyte depletion causes albuminuria. In the norm-albuminuria cohort (n = 75) baseline UPPod:CR was associated with development of albuminuria (*P* = 0.007) and, in the tertile with both normal kidney function (eGFR 84 ± 11.7 ml/min/1.73m^2^) and norm-albuminuria at baseline, UPPod:CR was associated with eGFR loss rate (*P* = 0.003). In type 2 diabetics with micro- or macro-albuminuria UPPod:CR and albuminuria were equally good at predicting eGFR loss. For norm-albuminuric type 2 diabetics UPPod:CR predicted both albuminuria and eGFR loss.

## Introduction

Diabetes accounted for 12.8% of global all-cause mortality among adults in 2015. Global prevalence of diabetes mellitus was estimated at 415 million in 2015 and is predicted to increase 55% by 2040. In parallel, total global health expenditure for diabetes estimated at 673 billion U.S dollars (12% of global health expenditure) in 2015 is expected to increase to 802 billion U.S dollars^1^. At the same time early intensive therapy in type 1 diabetes is reported to reduce risk of glomerular filtration rate decline by half emphasizing that very early identification of progression risk and intensive intervention can be expected to improve renal outcome and reduce costs^2^.

The classic clinical course of diabetic nephropathy proceeds through hyperfiltration leading to micro-albuminuria as an early indicator, and then progressing to albuminuria in about 10–20% of cases per year, and then to macro-albuminuria after about 10–15 years^3^. Once macro-albuminuria is present kidney function declines at about 2–20 ml/min per year ultimately causing end stage kidney disease (ESKD). High prevalence of diabetes makes diabetes-associated nephropathy the major cause of ESKD worldwide with its attendant human and monetary costs^1,3^. Progressive loss of kidney function may also occur in the absence of proteinuria in up to 30% of cases suggesting that alternative pathways to progression contribute^4–6^. Recently, markers of tubule-interstitial injury including L-type fatty acid binding protein (L-FABP), Kidney injury molecule-1 (KIM-1), neutrophil gelatinase-associated lipocalin (NGAL)^7–12^, oxidative stress marker (8-OHdG)^13^ and inflammatory marker (IL-6, IL-18, TNF-α) have been reported to be associated with progression in diabetic renal disease^14–18^ compatible with non-glomerular pathways participating in the progression process.

Degree of urinary albumin excretion above a threshold level is used for early diagnostic assessment of diabetic nephropathy progression risk^19^. Increased urinary albumin excretion represents the net difference between increased leak of albumin through the glomerular filtration barrier and the capacity of the proximal tubule to reabsorb filtered albumin from the tubular lumen. Thus both glomerular and tubular mechanisms can contribute to net albumin excretion, suggesting that albuminuria will tend to be an insensitive and non-specific marker at early stages although it will become more reliable as disease advances.

Podocyte depletion per se causes proteinuria and glomerulosclerosis, and persistent podocyte loss is a major factor driving glomerular disease progression^20–36^. Landmark studies in both type 1 and type 2 diabetes document the quantitative relationship between degree of podocyte depletion and amount of proteinuria and glomerulosclerosis^22,23,26^. Since podocytes are resident on the urinary space side of the glomerular basement membrane as they detach they can be identified in urine as markers for monitoring glomerular disease activity, progression and response to treatment in real time. We recently reported that urinary podocyte-specific mRNA markers precede albuminuria as an early marker of glomerulopathy in a Zucker rat model of type 2 diabetes^37^. Other investigators using alternative podocyte markers have shown correlation with progression in model systems and man. This includes counting viable podocytes in urine^38^, measuring podocyte-specific proteins in urine^39^, and measuring subcellular podocyte-derived exosomes and other subcellular fragments identified on the basis of their podocyte-specific protein expression^40,41^.

In this study we used a low speed urinary centrifugate designed to capture whole cells but not subcellular particles, analogous to that used to identify cells, casts and crystals in urine for diagnostic purposes. This low-speed urinary pellet is then processed to allow cell-specific RNA transcripts to be quantitated using TaqMan assays. This approach has the advantage that it is sensitive, quantitative and cell-specific because podocytes and other kidney cells express unique transcripts. As with all urinary assays, markers must be adjusted to allow for variation in urine concentration. We have used two approaches. First, we have expressed urinary mRNA markers per urinary creatinine concentration analogous to the urinary protein to creatinine ratio routinely used world-wide for spot estimation of protein excretion. Second, we have expressed kidney cell mRNA markers in relation to another RNA marker measured by the same method in the same sample at the same time. Since cells in urine can arise from anywhere in the urinary tract, this ratio approach cannot use common housekeeping markers such as GAPDH or beta-actin, but must use kidney-specific markers whose cellular origin is known. In the case of the podocyte we have used two podocyte-specific markers, podocin (NPHS2) and nephrin (NPHS1). Since nephrin mRNA is preferentially down-regulated under stress conditions while podocin mRNA is not, we have used the podocin:nephrin mRNA ratio (two markers from the same cell) as a measure of podocyte stress. Alternatively, in order to compare events happening in the glomerulus to events happening in the down-stream tubular compartment we have used the podocin to aquaporin 2 mRNA ratio, where aquaporin 2 is a marker of the distal tubule/collecting duct. We now have extensive experience with this approach in both model systems and man that has proven to be informative^30,31,35,42–48^.

To address the question of whether urinary podocyte markers can detect progression risk prior to albuminuria in type 2 diabetes as was suggested by rat modelling^37^, we first performed a cross-sectional study of urinary pellet podocyte mRNAs in a cohort of type 2 diabetics at various stages of nephropathy. As a second phase of this study, patients from phase 1 were then enrolled prospectively and followed for a period of 4 years to determine whether or not urinary podocyte or tubular mRNA markers measured at study onset would be predictive of renal function changes over the 4-year follow up period.

## Results

### Demographic information

Table 1A,B shows demographic profiles for norm-albuminuric, micro-albuminuric and macro-albuminuric type 2 diabetic groups in cross-sectional and longitudinal study (n = 165 and 124, respectively) and age and sex-matched healthy controls (n = 41). Notably, sex distribution that was skewed towards males in diabetic groups was matched by a similar skew in controls. Both systolic and diastolic blood pressure was significantly higher in diabetics compared to controls. eGFR (calculated by Japanese equation modified IDMS-MDRD study method^49^) was decreased in association with increasing albuminuria. Diabetic and anti-hypertensive medications used are shown. In this study, DPP4-inhibitors were used in a high proportion of patients reflecting Japanese practice^50^.

**Table 1:** (A) Demographics of patients in diabetes mellitus groups and the age-matched healthy control group. (B) Demographics of patients in diabetes mellitus groups in 4-year follow up longitudinal study.

	Control	DM Norm-albuminuric	DM Micro-albuminuric	DM Macro-albuminuric	*P* value
		Alb/Cre < 30	30 < Alb/Cre < 300	Alb/Cre > 300	
	(n = 41)	(n = 94)	(n = 36)	(n = 35)	
**(A)**					
Age (yrs)	63.8 ± 4.0	63.7 ± 9.0	65.4 ± 10.4	65.0 ± 11.3	0.748
Gender (M/F) (% male)	26 / 15 (63)	61 / 33 (65)	30 / 6 (83)	23 / 12 (66)	0.225
Body Mass Index (kg/m^2^)	21.9 ± 2.8	24.2 ± 3.8	25.4 ± 3.2	25.0 ± 3.5	< 0.001
Duration of diagnosed DM (months)	–	12.3 ± 10.2	12.0 ± 10.3	12.4 ± 9.6	0.992
Systolic Blood Pressure (mmHg)	120 ± 11	139 ± 16	149 ± 16	148 ± 13	< 0.001
Diastolic Blood Pressure (mmHg)	75 ± 7	80 ± 12	83 ± 15	82 ± 12	0.019
e-GFR (ml/min/1.73m^2^)	74.2 ± 13.4	69.7 ± 14.0	64.0 ± 18.6	51.7 ± 23.2	< 0.001
Fasting Blood Glucose (mg/dl)	95 ± 10	–	–	–	–
HbA1c (%)	–	7.0 ± 0.8	7.3 ± 0.8	7.1 ± 0.7	0.115
ACEi/ARB use (%)	0	23.4	66.7	60	–
Insulin use (%)	0	29.8	36.1	48.6	–
Biguanide use (%)	0	69.1	75	60	–
DPP4i use (%)	0	54.3	72.2	71.4	–
GLP1RA use (%)	0	6.4	11.1	8.6	–

	DM Norm-albuminuric	DM Micro-albuminuric	DM Macro-albuminuric	*P* value	
	Alb/Cre < 30	30 < Alb/Cre < 300	Alb/Cre > 300		
	(n = 75)	(n = 23)	(n = 26)		
(**B**)					
Age (yrs)	64 ± 9	65 ± 9	63 ± 11	0.897	
Gender (M/F) (% male)	48 / 27 (64)	18 / 5 (78)	15 / 11 (58)	0.303	
Body Mass Index (kg/m^2^)	24.1 ± 4.0	25.4 ± 3.4	25.8 ± 3.5	0.086	
Duration of diagnosed DM (years)	13.1 ± 10.5	12.3 ± 9.9	11.1 ± 7.7	0.667	
Systolic Blood Pressure (mmHg)	140 ± 16	150 ± 15	148 ± 14	0.008	
Diastolic Blood Pressure (mmHg)	79 ± 12	84 ± 14	83 ± 12	0.169	
e-GFR (ml/min/1.73m^2^)	68.8 ± 14.2	65.1 ± 17.4	54.0 ± 22.4	0.001	
HbA1c (%)	7.0 ± 0.8	7.0 ± 0.5	7.2 ± 0.7	0.466	
ACEi/ARB use (%)	25.3	78.3	61.5	–	
Insulin use (%)	33.3	30.4	50	–	
Biguanide use (%)	69.3	78.2	65.4	–	
DPP4i use (%)	53.3	82.6	69.2	–	
GLP1RA use (%)	8.0	4.3	11.5	–	

The data are given as mean ± 1SD. *Abbreviations:* e-GFR, estimated-glomerular filtration rate; ACE-I, angiotensin converting enzyme-inhibitor; ARB, angiotensin receptor blocker; DPP4i, Dipeptidyl Peptidase-4 inhibitor; GLP1RA, glucagon like peptide-1 receptor agonist.

### Urinary pellet podocyte and tubular mRNA markers were increased in diabetics

**Figure 1:**
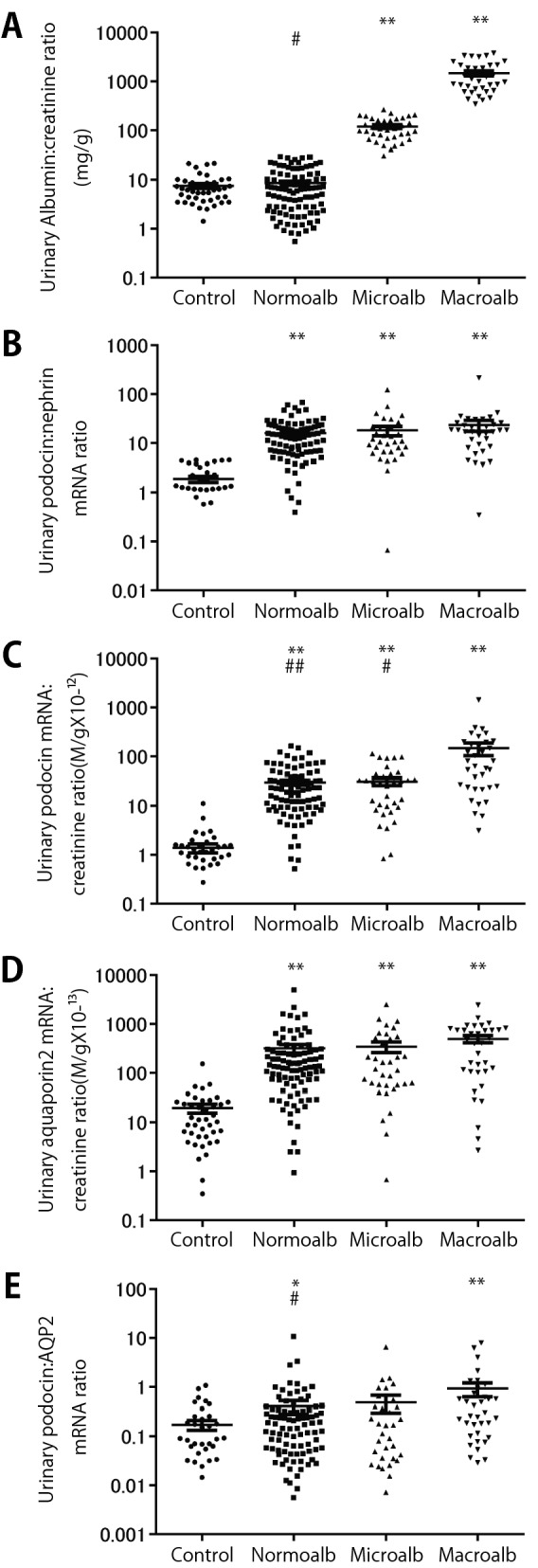
Urinary pellet podocyte mRNA markers and albuminuria in healthy control and at various stages of diabetic kidney disease. (**A**) Urinary albumin:creatinine ratio. (**B**) Urinary pellet podocin:nephrin mRNA ratio. (**C**) Urinary pellet podocin mRNA:creatinine ratio. (**D**) Urinary pellet aquaporin 2 mRNA:creatinine ratio. (**E**) Urinary pellet podocin:aquaporin 2 mRNA ratio. Urinary pellet excretion of podocyte-specific markers (measured using the podocin mRNA marker) and tubular marker (measured using the aquaporin 2 mRNA marker) were both increased in diabetics. These data are compatible with injury occurring to both glomerular and tubular compartments in diabetes. The macro-albuminuric group was losing podocytes more rapidly than tubular cells.*P < 0.05 and **P < 0.01 vs control, #*P* < 0.05 and ##*P* < 0.01 vs macro-albuminuria, assessed using the Kruskal–Wallis test followed by the Dunn test.

Figure 1A shows groupings by urinary albumin:creatinine ratio (UAlb:CR) that defines the group for subsequent analysis.

Figure 1B shows that the urinary pellet podocin:nephrin mRNA ratio (UPPod:Neph, podocyte hypertrophic stress marker) was significantly increased in all diabetics in the cohort regardless of degree of albuminuria as follows: norm-albuminuric (8.4-fold, *P* < 0.01), micro-albuminuric (9.6-fold, *P* < 0.01) and macro-albuminuric groups (12.4-fold, *P* < 0.01), respectively compared to controls. We interpret these data show that podocytes in diabetic glomeruli of all individuals in the cohort are experiencing stress regardless of the degree of albuminuria.

Figure 1C shows that the rate of podocyte detachment measured by the urinary pellet podocin mRNA:urinary creatinine ratio (UPPod:CR) was increased 21.6-fold (*P* < 0.01) in the norm-albuminuric group, 22.7-fold (*P* < 0.01) in the micro-albuminuric group and 105.3-fold (*P* < 0.01) in the macro-albuminuric group. We interpret these data to indicate that the rate of podocyte detachment in norm-albuminuric and micro-albuminuric groups were similarly increased while that in the macro-albuminuric group had a higher rate of detachment.

Figure 1D shows that the urinary pellet aquaporin 2 mRNA:urinary creatinine ratio (UPAqp2:CR, a tubular marker) was also increased 16.1-fold (*P* < 0.01) in the norm-albuminuric group, 17.6-fold (*P* < 0.01) in the micro-albuminuric group and 25.4-fold (*P* < 0.01) in the macro-albuminuric group. These data suggest that tubular injury as well as glomerular injury is taking place in all diabetic cohorts.

Figure 1E shows the ratio of urinary pellet podocin:aquaporin 2 mRNA ratio (UPPod:Aqp2, a measure of relative glomerular vs tubular injury) was not significantly increased above control in norm-albuminuric and micro-albuminurc groups but was increased above control in the macro-albuminuric group. The macro-albuminuric group was losing podocytes more rapidly than tubular cells indicating preferential glomerular vs tubular injury.

Taken together these data are compatible with injury occurring to both glomerular and tubular compartments in diabetes.

### 4-year prospective analysis

124 patients of the original 165 patients (75%) were available for analysis at the end of the four year follow-up.

### eGFR slope over 4 years of follow up

Using the 4-year eGFR values we estimated annualized eGFR slopes for each individual patient. In a univariable analysis (Table 2A) only UPPod:CR and UAlb:CR were significant predictors of the annualized GFR slope. In contrast, UPPod:Neph, UPAqp2:CR and UPPod:Aqp2 were not significantly related to eGFR slope. Therefore, among urinary pellet mRNA markers only UPPod:CR and UAlb:CR were used for multivariable analysis. In addition, we had a priori decided to use clinical variables including age, gender, baseline eGFR, HbA1c, mean arterial pressure and ACEi/ARBs use in the multivariate model. In the final multivariate model only UPPod:CR and UAlb:CR and not clinical factors predicted GFR decline (Table 2B).

**Table 2:** (A) Univariate analysis of the relationship of clinical and urinary markers with annualized slope of eGFR over 4-year follow up. (B) Multivariate analysis of the relationship of clinical and urinary markers with annualized slope of eGFR over 4-year follow up.

Variable	Coefficient	SE	*P* value	95% CI (LCL, UCL)
**(A)**				
Age (years)	− 0.03	0.31	0.36	− 0.90, 0.03
Males	− 0.51	0.62	0.42	− 1.75, 0.73
eGFR (ml/min/1.73m^2^)	− 0.007	0.17	0.66	− 0.04, 0.03
BMI (kg/m^2^)	0.04	0.08	0.65	− 0.12, 0.19
MAP (mm Hg)	− 0.01	0.02	0.66	− 0.06, 0.04
HbA1c (%)	− 0.39	0.41	0.35	− 1.20, 0.43
UAlb:CR (mg/gCre)	− 0.002	0.0004	** < 0.001**	− 0.002, − 0.0007
UPPod:CR (M/gCre)	− 0.02	0.005	** < 0.001**	− 0.03, − 0.01
UPAqp2:CR (M/gCre)	− 0.0009	0.0005	0.10	− 0.002, 0.0001
UPPod:Neph	− 0.02	0.01	0.16	− 0.04, 0.007
UPPod:Aqp2	− 0.31	0.20	0.12	− 0.70, 0.09
ACEi/ARB use	− 0.81	0.60	0.18	− 1.99, 0.37
Insulin use	− 0.46	0.62	0.46	− 1.68, 0.77
Biguanides use	0.06	0.65	0.93	− 1.24, 1.35
DPP4i use	− 0.83	0.61	0.18	− 2.04, 0.38
GLP1R use	0.23	1.14	0.84	− 2.03, 2.49
**(B)**				
Age (years)	− 0.06	0.03	0.09	− 0.125, 0.009
Males	− 0.94	0.59	0.11	− 2.110, 0.226
eGFR (ml/min/1.73m^2^)	− 0.03	0.02	0.09	− 0.071, 0.005
HbA1c (%)	− 0.39	0.39	0.31	− 1.150, 0.378
MAP (mm Hg)	− 0.008	0.02	0.72	− 0.053, 0.040
ACEi/ARB use	− 0.17	0.58	0.77	− 1.330, − 0.980
UPPod:CR (M/gCre)	− 0.01	0.005	**0.01**	− 0.024, − 0.003
UAlb:CR (mg/gCre)	− 0.002	0.0005	**0.004**	− 0.003, − 0.0005

**Figure 2:**
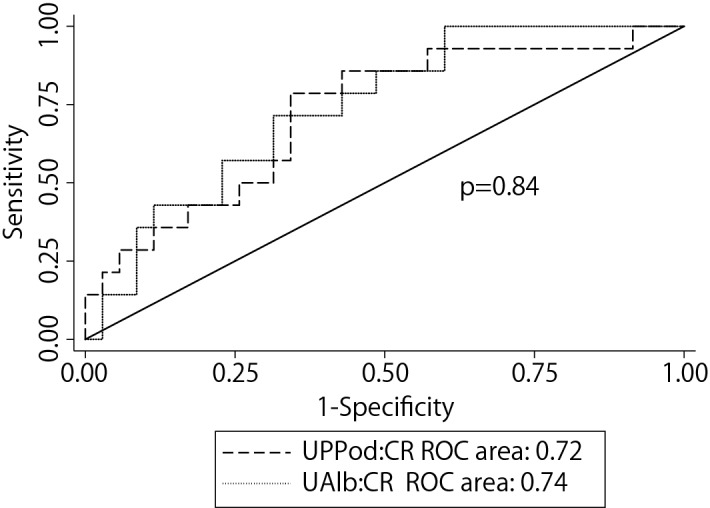
AUC analysis comparing albuminuria to urinary pellet podocyte mRNA excretion to predict decrease in eGFR over a 4 year follow-up period. No statistical difference was observed between these two markers. Thus once albuminuria is present it predicts rate of eGFR decline as well as, but no better than, the UPPod:CR.

Since accelerated podocyte detachment causes podocyte depletion which in turn causes proteinuria we anticipated that if the podocyte depletion hypothesis was correct that there would be overlap between the information provided by UAlb:CR and UPPod:CR. To test this concept, we calculated the predictive performance (AUC) to compare clinical parameters with or without the UAlb:CR and/or the UPPod:CR for their ability to predict a decrease in eGFR of ≥ 3 ml/min/1.73m^2^/year. Since AUC of a set of independent variables using all the cases from the original cohort tends to result in an overly optimistic estimation of AUC, we performed a K-fold cross validation (shown in Table 3 and Fig. 2). For this cohort followed for 4 years clinical data alone did not significantly predict progression. Both UAlb:CR and UPPod:CR alone improved the AUC to 0.67. Combining UAlb:CR and UPPod:CR minimally improved the AUC to 0.68. Combining clinical variables with both UAlb:CR and UPPod:CR marginally improved the AUC to 0.70. Thus, although UAlb:CR and UPPod:CR provided independent predictive information their combination did not significantly improve the predictive performance of the model.

**Table 3:** Comparison of Clinical variables alone with urinary markers alone or in combination to predict GFR decline of ≥ 3 ml/min/1.73m^2^/year using K fold cross validation.

Parameters	Cross-validated mean AUC	95% CI (LCL, UCL)
Clinical variables^#^	0.53	0.33, 0.64
UAlb:CR alone	0.67	0.52, 0.82
UPPod:CR alone	0.67	0.52, 0.81
UAlb:CR + UPPod:CR	0.68	0.55, 0.84
Clinical Variables^#^ + UAlb:CR	0.66	0.53, 0.82
Clinical variables^#^ + UPPod:CR	0.65	0.51, 0.77
Clinical variables^#^ + UAlb:CR + UPPod:CR	0.70	0.53, 0.82

^#^Clinical variables used are baseline eGFR, MAP, HbA1c and Age.

**Figure 3:**
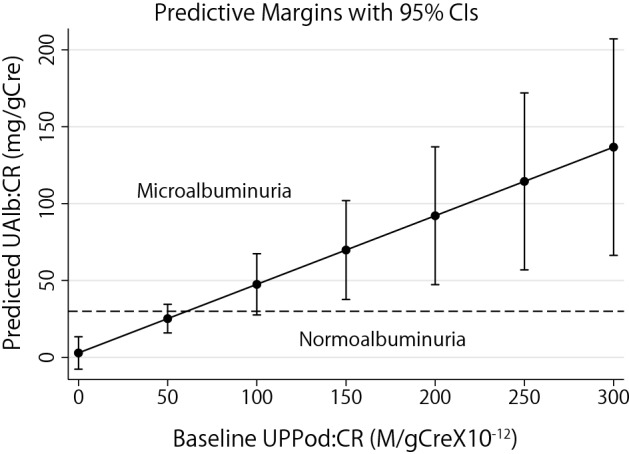
Relationship between baseline podocyte detachment (UPPod:CR) and the predicted UAlb:CR in normo-albuminuric patients adjusted for various clinical factors. The amount of UPPod:CR at baseline was significantly associated with increased albumin excretion rates (UAlb:CR) over the 4 year period of observation. The predictive margins were calculated using linear mixed models clustered at the patient level.

Data shown in Fig. 1 demonstrates that UPPod:CR was increased in the norm-albuminuric group (compared to controls), suggesting that increased UPPod:CR occurs at an earlier stage than micro-albuminuria and therefore might be a sensitive marker of progression in diabetes as had been observed in the rat type 2 diabetes model. Table 4A, B and Fig. 3 shows that when restricting the cohort to those with norm-albuminuria at baseline (n = 75) the amount of UPPod:CR at baseline was significantly associated with albumin excretion rates (UAlb:CR) over the 4 year period of observation (*P* = 0.001). This is compatible with increased podocyte detachment preceding development of increased urinary albumin excretion rates as observed in the Zucker rat model^37^.

**Table 4:** (A) Univariable Analysis: Relationship between various clinical factors, urinary mRNA markers with the albumin excretion rates over 4 years among those with normoalbuminuria at baseline (n = 75). (B) Multivariable Analysis: Relationship between baseline level of podocyte detachment that is adjusted for various clinical factors with the albumin excretion rates over 4 years among those with normoalbuminuria at baseline (n = 75).

Variable	Coefficient	SE	*P* value	95% CI (LCL, UCL)
**(A)**				
Age (years)	0.05	0.44	0.91	− 0.82, 0.92
Males	6.31	8.40	0.45	− 10.16, 22.78
HbA1c (%)	− 8.30	4.94	0.09	− 17.99, 1.38
MAP (mm Hg)	0.23	0.33	0.48	− 0.42, 0.88
eGFR (ml/min/1.73 m^2^)	− 0.16	0.26	0.54	− 0.67, 0.35
BMI (kg/m^2^)	2.09	1.01	**0.04**	0.11, 4.07
ARB or ACEi use	1.97	8.48	0.82	− 14.566, 18.60
UPPod:CR (M/gCre)	0.33	0.12	**0.007**	0.09, 0.57
UPPod:Neph	− 0.38	0.35	0.28	− 1.08, 0.31
UPPod: Aqp2	1.86	4.63	0.69	− 7.22, 10.95
**(B)**				
UPPod:CR (M/gCre)	0.47	0.14	**0.001**	0.20, 0.73
Age (years)	− 0.27	0.52	0.98	− 0.93, 0.90
Males	14.41	8.77	0.1	− 2.77, 31.61
BMI (kg/m^2^)	3.10	1.06	**0.003**	1.02, 5.19
MAP (mm Hg)	0.11	0.34	0.74	− 0.56, 0.78
HbA1c (%)	− 7.14	4.92	0.15	− 16.79,2.51
eGFR (ml/min/1.73 m^2^)	− 0.35	0.30	0.24	− 0.93, 0.23
ARB or ACEI use	− 4.19	8.75	0.63	− 21.34, 12.94

**Figure 4:**
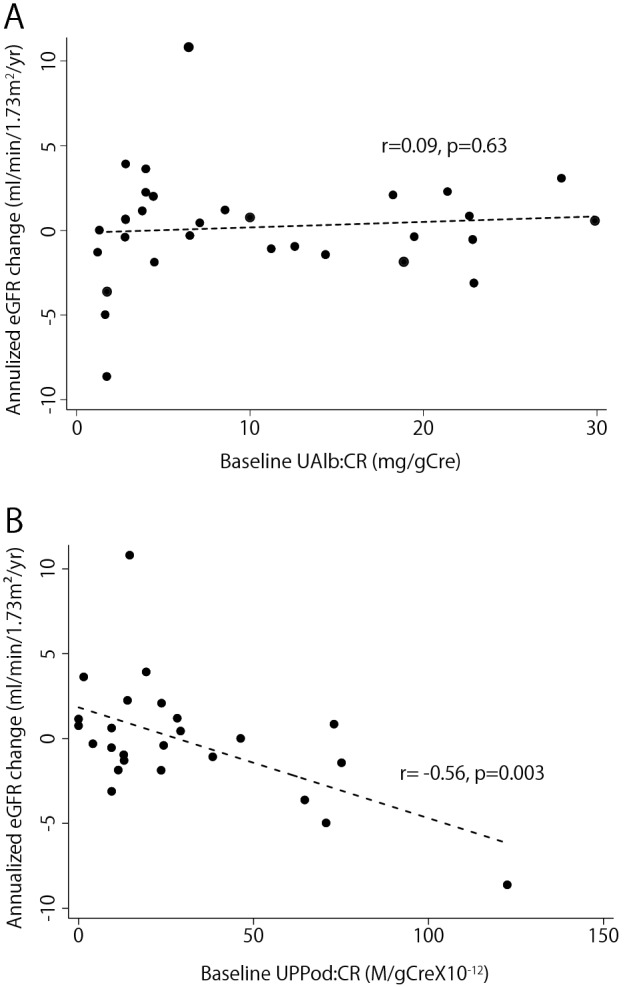
Relationship between baseline level of podocyte detachment (UPPod:CR) (**A**) and albuminuria (UAlb:Cre) (**B**) and GFR decline among those with normoalbuminria at baseline in the tertile with eGFR. In this group UAlb:CR within the normal range was not associated with eGFR decline (r = 0.09, *P* = 0.63). In contrast, UPPod:CR was significantly associated with eGFR decline (r =  − 0.56, *P* = 0.003) in the highest tertile of baseline eGFR (n = 25, Mean eGFR = 84 ± 11.7 ml/min/1.73 m^2^, range 72–127 ml/min/1.73 m^2^).

As a next step we tested whether baseline levels of UPPod:CR and UAlb:CR among those who were norm-albuminuric was associated with kidney disease progression as measured by the eGFR slope. To focus on early diabetic glomerular disease, we limited analysis to those in the highest tertile of baseline eGFR (n = 25, Mean eGFR = 84 ± 11.7 ml/min/1.73 m^2^, range 72–127 ml/min/1.73 m^2^). In this group UAlb:CR within the normal range was not associated with eGFR decline (r = 0.09, *P* = 0.63) (Fig. 4A). In contrast, UPPod:CR was significantly associated with eGFR decline (r =  − 0.56, *P* = 0.003) (Fig. 4B). In parallel analyses we examined norm-albuminuric diabetics with decreased eGFR at baseline and found no relationship of the eGFR slope with any urinary markers, including the tubular marker aquaporin 2 (data not shown).

Taken together these data are consistent with increased UPPod:CR (podocyte detachment) identifying early risk for progression as defined by both development of albuminuria and eGFR decline.

## Discussion

In this report we assessed whether or not urinary pellet cell-specific mRNAs for podocytes might serve as early markers for detecting progression in a cohort of type 2 diabetics. The study was presaged by the observation that urinary pellet podocyte　markers increased significantly prior to onset of albuminuria in a model of type 2 diabetes-associated nephropathy in the Zucker rat^37^. As was observed in the rat model system we found that humans with type 2 diabetes had evidence of hypertrophic podocyte stress (increased UPPod:Neph) and had higher rates of podocyte detachment into the urine (reflected by UPPod:CR). However, we also found that the distal tubular/collecting duct marker (UPAqp2 mRNA) was increased above control in both albuminuric and non-albuminuric diabetics, suggesting that both tubular injury and podocyte injury were occurring at an increased rate. To determine whether or not the markers would provide predictive information, we followed the cohort for 4 years and then examined the relationship between the rate of loss of eGFR over the 4 years and the markers measured in the baseline sample. We found that neither the podocyte stress marker (UPPod:Neph) nor the tubular marker (UPAqp2:CR) predicted loss of eGFR in the cohort. In contrast, UPPod:CR did predict rate of eGFR loss, as had been observed in the rat model.

The podocyte depletion hypothesis posits that podocyte depletion due to accelerated loss and/or glomerular volume enlargement creates podocyte hypertrophic stress such that podocyte foot processes are not able to fully cover the filtration surface area resulting in leak of albumin across the filtration barrier. If this scenario is correct then we would expect that increased rate of podocyte detachment would eventually lead to albuminuria that over time would increase causing glomeruli to adapt by scar formation in an attempt to reduce the protein leak eventually causing glomerulosclerosis, nephron loss and reduction in eGFR. Thus, the observation that in diabetics with micro- and macro-albuminuria the rate of podocyte detachment paralleled the degree of albuminuria was the expected finding. Indeed, AUC analysis showed that the capacity of UPPod:CR and UAlb:CR for predicting a rate of eGFR loss of greater than 3 ml/min/1.73m^2^/year overlapped. Thus, in already albuminuric diabetics these two markers provide similar levels of predictive information.

The question at issue is whether or not the UPPod:CR marker might be more sensitive than albuminuria at early stages of diabetic renal injury as had been the case for the rat model and had been suggested by the cross-sectional phase1 part of the study showing that UPPod:CR was already increased in norm-albuminuric diabetics. Two pieces of data from this report suggest that this is the case. First, the baseline UPPod:CR was significantly increased in this non-albuminuric cohort. Second, even in those norm-albuminuric diabetics in the upper tertile of renal function at baseline, UPPod:CR was strongly associated with reduction in eGFR over 4 years.

In this study, although the aquaporin 2 mRNA tubular marker was increased in the baseline cohort it was not significantly associated with eGFR decline over the 4 year follow-up, even in a cohort selected to represent norm-albuminuric progressors (decreased eGFR at baseline and loss of renal function during follow-up). We chose to examine this aquaporin 2 marker because it represents the most distal part of the tubule for comparison to the podocyte at the most proximal part of the tubule measured in the same urine pellet sample at the same time by the same method. We reasoned that significant upstream events impacting tubules such as nephron death would also be expected to accelerate detachment of aquaporin 2-containing cells. This result does not mean that markers of proximal tubule would not provide similar information as is suggested by other studies using NGAL or KIM-1 as tubular markers^9–12^. Large longer-term multicenter study will be required to compare tubular and podocyte markers for their capacity to predict progression in norm-albuminuric diabetics.

A limitation of this study is the relatively short follow-up time of 4 years. Longer term follow-up of this cohort and an independent larger cohort will be necessary to confirm this result although the data observed closely parallels our previous report in a Zucker rat model of type 2 diabetic nephropathy^37^. Another weakness of the study was that the urinary marker measurements were based on a single spot urine sample. We expect that, as with UAlb:CR as a predictor, serial urinary pellet podocyte mRNA markers would provide a more robust readout for an individual. Future studies will need to build in this requirement. Never-the-less in spite of these weaknesses that would tend to reduce the chances of significant relationships being observed, we found strong correlations between the urinary podocyte detachment and outcome in diabetes that mirror similar studies done in other human glomerular diseases and in model systems^24,27,30–37,42–48^.

In summary, we have demonstrated that urinary pellet podocyte mRNA excretion was as good as albuminuria in predicting rate of eGFR decline in diabetics with albuminuria. Furthermore, we show that the urinary pellet podocyte mRNA marker was increased even in norm-albuminuria diabetics, and predicted decreased eGFR in the 4 years of follow-up. These data suggest that urinary pellet excretion of podocyte mRNAs could potentially provide early diagnostic and progression prediction information in diabetic nephropathy to complement and extend conventional markers.

## Methods

### Ethical considerations

The human studies were conducted according to the principles contained within the Declaration of Helsinki and were approved by the Institutional Review Board of the University of Miyazaki Hospital (No.2014–055), Oita University Hospital (No.1374), Heiwadai Hospital (No.261121) and Nankai Medical Center (No.NC-H30-001). Informed consent was obtained from all subjects including healthy controls.

### Sample collection from the type 2 diabetic cohort

For this analysis type 2 diabetes was defined as all remaining adult diabetics excluding the type 1 juvenile onset cohort and those with anti-glutamic acid decarboxylase (GAD) and insulinoma-associated antigen-2 (IA-2) antibodies. From January 2015 to June 2015, spot urine samples from out-patient clinic were obtained from 165 consecutive patients at various stages of type 2 diabetes mellitus (norm-albulinuric group: n = 94, micro-albuminuric group: n = 36, macro-albuminuric group: n = 35) and age and sex-matched 41 healthy controls who did not have diabetes mellitus and/or hypertension in their general medical checkup. 124 patients from the original 165 patients (norm-albuminuric group: n = 75, micro-albuminuric group: n = 23, macro-albuminuric group: n = 26) were available for enrollment for 4-year follow up study. eGFR was estimated by the IDMS-MDRD method adjusted for the Japanese population (194 × Serum creatinine^−1.094^ × Age^−0.287^ × 0.739 [if female])^49^. Attrition in this 4 year cohort was due to the patient moving to another area (n = 32), death from non-kidney disease (n = 8) and withdrawn consent (n = 1). The clinical profile of patients with DM and age and sex-matched healthy controls are shown in Table 1 and 2. Urinary albumin:creatinine ratio (UAlb:CR), urinary pellet podocyte (podocin) mRNA factored urinary creatinine concentration (UPPod:CR, podocyte detachment marker) and urinary pellet aquaporin 2 mRNA factored urinary creatinine concentration (UPAqp2:CR, tubular injury marker) were measured. In addition, the urinary pellet ratio of two podocyte-specific markers podocin and nephrin mRNAs (UPPod:Neph, podocyte hypertrophic stress marker), and urinary pellet podocin mRNA factored aquaporin 2 mRNA ratio (UPPod:Aqp2, relative glomerular vs tubular injury marker) were measured. Furthermore, we performed a prospective observational cohort study of these participants using data from a 4-year follow-up period (2015–2019). Renal outcome was defined as a decrease in the estimated glomerular filtration rate (eGFR) loss of 3 ml/min/1.73m^2^ per year (detail of the analysis is provided in statistical analysis section).

### RNA from human urine sediments

Spot urine samples were collected at outpatient clinic visit. All urine samples were centrifuged at 4 °C for 15 min at 3200 × *g* on a tabletop centrifuge. The supernatant was removed, the pellet suspended in 1.5 mL diethyl pyrocarbonate-treated phosphate-buffered saline, and then centrifuged at 12 000 × *g* for 5 min at 4 °C. The washed pellet was resuspended in RLT/β-mercaptoethanol buffer (RNeasy kit; Qiagen, Germantown, MD, USA) and then frozen at –80 °C until RNA extraction^35,45^.

### RNA preparation and qRT-PCR assay

The total urinary pellet was purified using an RNeasy mini kit (cat. No. 74106; Qiagen). cDNA was transcribed from sample total RNA using a high-capacity cDNA reverse transcription kit (Applied Biosystems, Foster City, CA, USA). Quantitation of podocin, nephrin and aquaporin2 mRNA abundance was performed with a LightCycler 96 system (Roche Molecular System, Mannheim, Germany) using FastStart Essential DNA Probe Master Mix (Roche Molecular System, Inc.) in a final volume of 10 μL per reaction. The TaqMan probes (Applied Biosystems) used were: human NPHS1 (nephrin) (cat. No. Hs00190446_m1); human NPHS2 (podocin) (cat. No. Hs00922492_m1) and human aquaporin 2 (cat. No. Hs00166640_m1). All data were from 2-μg samples of cDNA measured in duplicate. cDNA standard curves were constructed using these serially-diluted standards, as previously described^35,45^.

### Statistical analysis

Statistical analysis of cross-sectional study was performed using GraphPad PRISM software, version 6.0 (GraphPad Software, Inc., La Jolla, CA, USA). Clinical profile of patients with DM are mean ± SD. Urinary measurements are mean ± SEM. Differences among the two groups were tested using the Mann–Whitney U test, and more than two groups were tested using the Kruskal–Wallis test. When the result of the Kruskal–Wallis test was significant, a Dunn test was performed for post hoc analysis. A *P* value < 0.05 was considered statistically significant.

For GFR slope analysis we performed a linear regression to obtain a “slope” of eGFR over the four years and divided this value by 4 to obtain and “annualized” rate of eGFR change. K-fold cross-validation studies were performed using the command cvAUROC a third party command^51^. For testing the association between baseline podocyte detachment and albuminuria in patients who were normoalbuminuric at baseline we used linear mixed models clustered at the patient level. Both univariable and multivariable analysis adjusted for multiple baseline clinical variables were performed. Predictive margins were calculated after the regression command to test the effect of increased baseline UPPod:CR on albuminuria keeping all other covariates constant. The “margins” and “marginsplot” command were used to generate margins ad plots of those margins. All longitudinal analysis was performed using Stata 15 I/C (college Station, TX)^52,53^.
